# Entropy of difference works similarly to permutation entropy for the assessment of anesthesia and sleep EEG despite the lower computational effort

**DOI:** 10.1007/s10877-024-01258-8

**Published:** 2024-12-26

**Authors:** Alexander Edthofer, Dina Ettel, Gerhard Schneider, Andreas Körner, Matthias Kreuzer

**Affiliations:** 1 Institute of Analysis and Scientific Computing, TU Wien, Wiedner Hauptstraße 8-10, 1040 Vienna, Austria; 2 Department of Anesthesiology and Intensive Care, School of Medicine and Health, Technical University of Munich, Ismaninger Str 22, 81675 Munich, Germany

**Keywords:** Anesthesia, Monitoring, Electroencephalogram, Entropy of difference, Permutation entropy

## Abstract

EEG monitoring during anesthesia or for diagnosing sleep disorders is a common standard. Different approaches for measuring the important information of this biosignal are used. The most often and efficient one for entropic parameters is permutation entropy as it can distinguish the vigilance states in the different settings. Due to high calculation times, it has mostly been used for low orders, although it shows good results even for higher orders. Entropy of difference has a similar way of extracting information from the EEG as permutation entropy. Both parameters and different algorithms for encoding the associated patterns in the signal are described. The runtimes of both entropic measures are compared, not only for the needed encoding but also for calculating the value itself. The mutual information that both parameters extract is measured with the AUC for a linear discriminant analysis classifier. Entropy of difference shows a smaller calculation time than permutation entropy. The reduction is much larger for higher orders, some of them can even only be computed with the entropy of difference. The distinguishing of the vigilance states between both measures is similar as the AUC values for the classification do not differ significantly. As the runtimes for the entropy of difference are smaller than for the permutation entropy, even though the performance stays the same, we state the entropy of difference could be a useful method for analyzing EEG data. Higher orders of entropic features may also be investigated better and more easily.

**Supplementary Information:**

The online version contains supplementary material available at 10.1007/s10877-024-01258-8.

## Introduction

Electroencephalographic recordings (EEG) during surgical anesthesia serve the purpose of monitoring the patient and aid the anesthesiologist in adjusting the anesthetic level. In the past years a number of analytical methods were used to extract EEG information that corresponds with the hypnotic component of anesthesia. Commercially available devices, so called "*depth-of-anesthesia-monitors*" process the EEG with spectral methods [1–4]. They utilize the changes of EEG frequencies induced by (mainly) GABAergic substances from faster rhythms during wakefulness to slower oscillations during anesthetic levels [5]. Apart from the spectral approaches, entropic parameters that analyze the EEG in the time domain gained increasing popularity in assessing the hypnotic component of anesthesia. The first parameter introduced to the anesthesia field was the approximate entropy [6–8]. Encouraged by the results of this parameter to distinguish different levels of anesthesia, a number of different entropic parameters were applied to EEG traces recorded during anesthesia. The most efficient parameter regularly used is the permutation entropy (PeEn) [9–12]. This symbolic parameter was designed by Bandt and Pompe [9] and introduced to anesthesia research by Jordan et al. and Olofsen et al. [8, 10]. Other research groups showed the high performance of this parameter as well [12]. PeEn creates a series of ordinal patterns from the EEG and uses the probability distribution of these ordinal patterns to calculate its value that corresponds with the complexity of the time series, i.e., the EEG according to the Shannon entropy [13]. Although the parameter shows good performance in evaluating the anesthetic level, the claim made of PeEn being a measure of complexity has been questioned [14]. In terms of anesthesia monitoring, i.e., to separate different levels of anesthesia, subparameters of PeEn showed even better performance [15]. When using PeEn, an embedding dimension *m* has to be defined that corresponds to the length of the EEG segment from which one ordinal pattern is derived. Berger et al. described an association of PeEn with $m=3$ to the spectrum of the EEG [14]. This $m=3$ setting also seems to function for anesthesia monitoring as well, as do higher *m* [8, 10]. Because the number of possible patterns is *m*!, PeEn calculation time strongly increases for higher *m*, but different approaches lead to different results [16]. Pasquale Nardone introduced another symbolic parameter, the *entropy of difference* (EoD), to evaluate the "*complexity*" of a time series [17]. He states that EoD is as efficient as PeEn, but it reduces the sample size to estimate the parameter value. He also states that for random signals, PeEn will lead to a constant probability of $q_m(\pi )=\frac{1}{m!}$ for a pattern $\pi$. This impedes the assessment of the "distance" between the probability found in a signal, i.e. $p_m(\pi )$, which is the EEG in our case, to a random signal, which would be white noise [17]. EoD has already proven useful to assess differences in sleeping behavior in an animal model for Alzheimer’s disease [18]. In this article, we compared the performance of EoD versus PeEn to assess sleep stages and anesthesia levels. With these analyses, we intended to investigate two features:

(i) *Can we further reduce the information content from the EEG used to assess vigilance states without sacrificing performance?*
(ii) *How does the change from ordinal (PeEn) to difference patterns (EoD) influence computing times, especially at higher embedding dimensions?*

## Methods

The data sets analyzed and the entropy-based parameters are described at the beginning of this section. The algorithms used to compute them and their impact on the runtime are then presented. The statistical analysis concludes this section.

### Included studies

#### CAP sleep database

For our sleep analysis, we used an openly available data set, the cyclic alternating patterns (CAP) sleep database [19], which is available on the physionet.org site [20]. This data bank contains 105 polysomnography recordings, 91 of them have different sleep disorders, and 14 are datasets of people who do not suffer from a neurological or other sleep-related disorder. The last group also did not take any medication affecting the central nervous system. They were recorded at the Sleep Disorders Centre of the Ospedale Maggiore di Parma, Italy. The breakdown of the recordings regarding the different diseases of the subjects is given in [19]. As we were interested in comparing the performance features of PeEn to EoD, we did not separate the dataset by disease, but only investigated the differences in the parameters at the different sleep stages. The records from the database contain at least two EEG channels, two channels with electrooculographic (EOG) data, two channels with electromyographic (EMG) data, electrocardiographic (ECG) data, and respiratory signals as well as the subjects’ age. They also contain the sleep-scoring vectors that enable the assignment of a vigilance state to an EEG episode. Because of our analytical intention, we only used the EEG information. The EEG was recorded from channels placed according to the 10-20 system. If available, we considered the recordings of channels Fp2-F4 or F2-F4, which was the case in 98 out of 105 recordings. In the other cases, a centrally located channel was chosen. For standard EEG pre-processing, we set a low-pass filter of 30 Hz at a sampling frequency of 200 Hz [21]. The classification of the sleep stages was carried out according to the R&K rules [22]. For our purpose, we used the modern classification rules according to AASM [23]. Therefore, we merged sleep stages S4 and S3 into the combined non-rapid-eye-movement sleep (NREMS) stage 3. So we included the stages wake (W), REMS, NREMS1, NREMS2, and NREMS3.

#### Anesthesia data

To evaluate the performance of PeEn and EoD at different levels of anesthesia, we retrospectively analyzed data from a published study from 2009 [24] that was designed to evaluate a combined method of EEG and auditory evoked potentials examination to detect the consciousness state during anesthesia. The details of, e.g., the anesthesia protocol can be found in the original publication [24]. In short, EEG was recorded from positions M2 and AT1, the latter one is at the left temporal region between the lateral edge of the eye and upper edge of the ear, with Fz as the common reference and Fpz as the ground electrode with four ZipPrep electrodes (Aspect Medical Systems, Natick, MA) using a device made for intraoperative recordings of EEG and evoked potentials. Digitization happened with a sample rate of 1 kHz and a high-pass filter of 0.5 Hz and a low-pass filter of 400 Hz were applied using the software tool NeuMonD [25] which is based on LabView™. We used EEG data that was recorded during wakefulness before anesthesia, burst suppression phases, and a light (inter1) and deep anesthesia level (inter2). For our analyzes, the EEG was resampled at 100 Hz and a low pass filter of 30 Hz was applied.

### Entropy-based EEG analysis

Entropic parameters can be used to differentiate the EEG recorded from different vigilance states during sleep [26, 27] or anesthesia [8, 10, 12]. In particular, PeEn was identified as a suitable candidate for separating unconscious from conscious states [8, 10, 28]. Hence, we used both PeEn and its modification, EoD, in our analyses. EoD is also based on the Shannon entropy [13] but uses patterns of the sign of the difference between two data points [17] instead of ordinal order patterns [9].

The parameters are calculated of the time series $(x_t)_{t \in I}, I = \{1,\ldots ,N\},$ with a certain length *N*, which represents the EEG signal. First, an embedding dimension or order *m* and time-delay $\tau$ are chosen, and the series is split into

1 $$\begin{aligned} k := N-(m-1) \tau \end{aligned}$$

tuples of length *m*. The time delay indicates the index shift between two values in the tuple, i.e., for $\tau =1$, the values in the tuple are also neighboring values in the time series. For the sake of simplicity, we set $\tau = 1$, as the influence of the time delay is already discussed by Popov et al. in a manuscript [29].

#### Permutation entropy

**Fig. 1:**
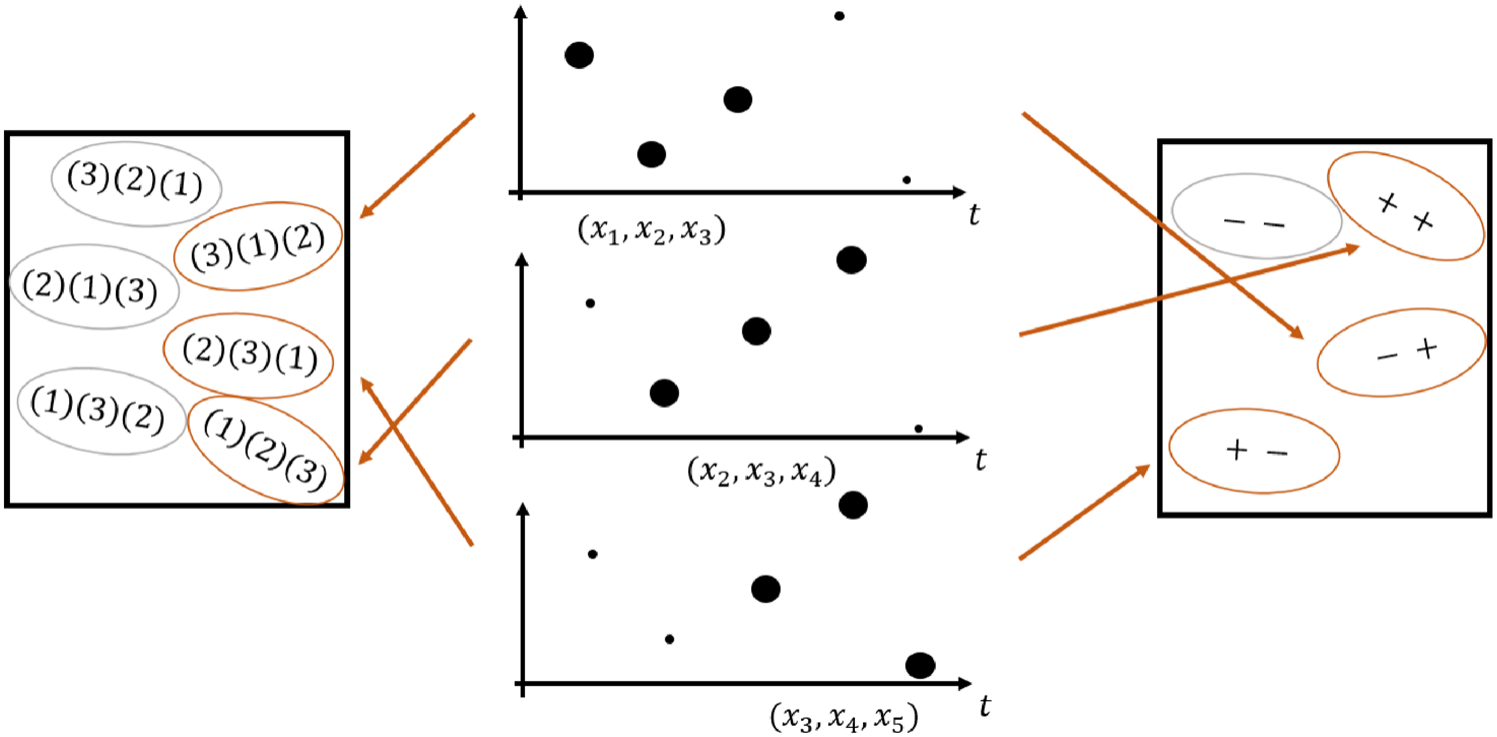
The PeEn and the EoD break the EEG time series down in specific patterns to analyze the signal further in the time domain. In the middle you can see an example time series encoded once for the PeEn on the left side and once for the EoD on the right side. The numbers in the left box present the rank order of amplitudes, with the highest number being the highest amplitude in the pattern, that are used for PeEn calculation. The signs in the right box represent the sign pattern reflecting the difference in amplitude (higher of lower) of the next amplitude value of the pattern. These sign patterns are used for EoD calculation

The publications of Bandt and Pompe or Jordan et al. provide a detailed description of the PeEn algorithm [8, 9]. The *k* tuples of length *m* form the base for the order coding. The highest amplitude within this segment is denoted as rank *m*; the second highest amplitude is denoted as rank $m-1$, etc. The lowest amplitude has rank 1. This leads to *m*! possible rank patterns (ordinal patterns) $\pi _i$ or permutations with *i* ranging from 1 to *m*!. Fig. 1 shows the mapping between the tuples of the time series and the ordinal patterns for a small example with $m=3$. With the classical formula to calculate the entropy according to Shannon [13], where $p(\pi _i)$ is the probability of occurrence of permutation type *i*, PeEn is defined as

2 $$\begin{aligned} PeEn=-\sum _{i=1}^{m!}{p(\pi _i)\log {p}(\pi _i)}. \end{aligned}$$

The base of the logarithm is usually 2. The range of the PeEn is $[0,\log (m!)]$ [13]. The minimal value 0 is reached, if there is one ordinal pattern, that is present for every tuple, i.e. for one pattern *j* it holds $p(\pi _j)=1$. Hence, all the other patterns never appear, i.e. $p(\pi _i)=0$ for all $i\ne j$, which results in $PeEn = 0$. The maximal value $\log (m!)$ is obtained, if the permutation type occurrence is evenly distributed, i.e. the probability of each ordinal pattern is $p(\pi _i) = \frac{1}{m!}$. This results in $PeEn = - m!\frac{1}{m!}\log \left( \frac{1}{m!} \right) = \log (m!)$. Therefore, a normalized modification of the PeEn can be obtained by dividing the measure by its maximal value,

3 $$\begin{aligned} {\widehat{PeEn}}=-\frac{1}{\log (m!)}\sum _{i=1}^{m!}{p(\pi _i)\log {p}(\pi _i)}. \end{aligned}$$

This way, the PeEn has a range of [0, 1] independent of *m*, which allows a comparison of the values between different orders.

In order to reliably calculate PeEn the numbers of tuples should at least be the number of possible permutations, i.e. $k\ge m!$. Otherwise the value of the PeEn cannot reach its maximum. Hence, the length of the time series should be $N\ge m!+(m-1)\tau$ [30, 31]. As we set $\tau =1$, we sum up the inequality to $N > m!+m$. This means, for a time series with 10 s at a sampling rate of 250 Hz the maximal order should be $m=6$. For comparable results and feasible occurrences of the different ordinal patterns, and because $m-1$ is always much smaller than *m*!, we simplify the inequality such that *N* should always be much larger than *m*! to

4 $$\begin{aligned} N \gg m!. \end{aligned}$$

#### Entropy of difference

Pasquale Nardone gives a detailed explanation of EoD in his paper [17]. The idea of EoD is that the differences between neighboring amplitude values in the time series define the entropy value, not ordinal patterns. Taking the segments of length *m* cut from the EEG representing time series $(x_t)_{t \in I}$, for calculating the EoD only the series of differences between consecutive values is of interest. Considering the *m*-tuple [1, 2, 4, 1], then the differences between the values are $[1, 2, -3]$. For EoD only the sign of the difference is relevant, i.e., $[+, +, -]$. This leads to $2^{m-1}$ possible combinations $\delta _l$ of "$+$" and "−" with *l* ranging from 1 to $2^{m-1}$. For a small example with $m=3$, Fig. 1 shows the mapping between the time series tuples and the difference patterns. The EoD is then given by

5 $$\begin{aligned} EoD=-\sum _{l=1}^{2^{m-1}}{p(\delta _l)\log { p}(\delta _l)}. \end{aligned}$$

Analogously to before, the base of the logarithm is usually 2 and the range is $[0,\log (2^{m-1})]$ [13], which can be reduced to $[0,m-1]$. The minimal value 0 is again obtained if one pattern appears all the time and the maximal $m-1$ if the patterns of differences have an equally distributed occurrence. The normalized measure of the EoD with a range of [0, 1] is, for a logarithm with base 2, given by

6 $$\begin{aligned} {\widehat{EoD}}=-\frac{1}{m-1}\sum _{l=1}^{2^{m-1}}{p(\delta _l)\log {p}(\delta _l)}. \end{aligned}$$

The possible patterns for the EoD are much fewer than for the PeEn. For a reliable calculation, we demand a number of tuples of at least the number of possible patterns, i.e., $k\ge 2^{m-1}$. With $\tau =1$, it follows that $N > 2^{m-1}+m$, from which arises that for a signal of 10 s with a sampling rate of 250 Hz, the maximal order can be $m=12$. This means, that the EoD can be computed with much higher orders than the PeEn. However, as we again want that every pattern of difference can occur equally frequently, such that all variations are feasible, we suggest that

7 $$\begin{aligned} N\gg 2^{m-1}. \end{aligned}$$

**Fig. 2:**
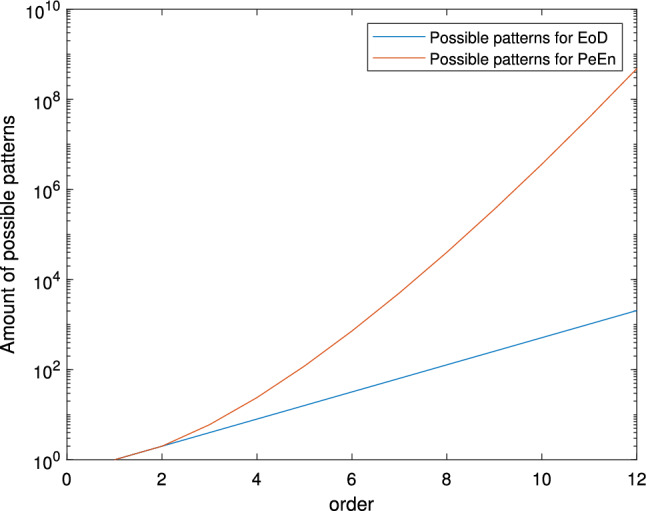
Number of possible patterns for the PeEn and the EoD for orders up to $m=12$

When the number of possible patterns is compared, one can see that for increasing *m*, the number of possible permutations $\pi _i$ rises stronger with *m*! possibilities than for $\delta _l$ with $2^{m-1}$ possible combinations for the patterns of difference. Fig. 2 shows the course of the possible combinations for increasing orders up to $m=12$.

### Encoding patterns

The need to identify and count the different patterns in a time series asks for a method to encode given data into a vector of pattern representations. Enumerating patterns with integers from 1 to *m*! or 1 to $2^{m-1}$ respectively allows for compact storing and simple comparison and makes counting patterns easy [16]. Regarding ordinal patterns, different ways of encoding are discussed [14, 32, 33]. In a paper by Berger et al. from 2019 the so-called Lehmer code is proposed for this purpose [16]. We will base this comparison on the results obtained there. In the case of difference patterns, interpreting the "$+-$"string as a binary representation of a natural number provides an intuitive encoding. We formally define these encoding methods and discuss different algorithms for implementing them.

#### Encoding ordinal patterns

The Lehmer code exploits the fact that the ordinal pattern of a tuple $(x_1, \dots , x_m)$ is uniquely identified by the tuple of the right inversion counts $(r_1, \dots ,r_m)$ where $r_i$ is defined as

8 $$\begin{aligned} r_i:= \sum _{j=i+1}^{m} [x_i > x_j] . \end{aligned}$$

The [.] operation has either 1 as the output if the statement is true or 0 if it is false. As $r_i$ is an integer in $\{0, \dots , m-i \}$, the sequence $(r_1) (r_2) \dots (r_m)$ can be interpreted as an *m*-digit factoradic numeral. Consequently, the numerical representation of the ordinal pattern of a tuple $(x_1, \dots , x_m)$ is given by the equation

9 $$\begin{aligned} n = \sum _{i=1}^{m-1} \left( (m-i)! \sum _{j=i+1}^{m} [x_i > x_j] \right) . \end{aligned}$$

Given a data vector that should be encoded in a vector of ordinal patterns of order *m*, a straightforward algorithm takes each *m*-tuple and calculates the representation in Lehmer code. Calculating $(m-i)!$ for each *i* in the sum can be avoided. The computational complexity for calculating one pattern representation is ${\mathcal {O}}(m^2)$, which gives a total computation time of ${\mathcal {O}}(N m^2)$. This is the *Plain Algorithm* described by Berger et al. [16]. The detailed calculation of the computational complexity and the pseudocode of the algorithm are in supplemental section S1.

This result can be improved by using the fact that knowing one pattern narrows down the possible patterns in the next step. Two adjacent tuples $(x_1, \dots , x_m)$ and $(x_2, \dots , x_{m+1})$ overlap in $m-1$ values. The right inversion counts of the new tuple can be calculated from the right inversion counts of the old tuple and the comparison of each value in $(x_2, \dots , x_m)$ to $x_{m+1}$. Therefore only $m-1$ comparisons are necessary. The algorithms *Overlap Algorithm* and *Lookup Algorithm* by Berger et al. implement this idea [16]. Since the lookup table would occupy more than 3GB of memory for orders $m \ge 10$, we used the overlap algorithm for our purpose. However, the two algorithms had very similar runtimes for low *m*. In both cases the computational complexity of encoding one ordinal pattern is reduced to ${\mathcal {O}}(m)$, and hence for the whole time series ${\mathcal {O}}(N m)$ [16]. The pseudocode of the algorithm and the detailed calculation of the computational costs can be found in supplemental section S1.

#### Encoding difference patterns

Each difference pattern of a tuple $(x_1, \dots , x_m)$ can be interpreted as the binary representation of a natural number. Following this idea, a numerical pattern representation in $\{ 1, \dots , 2^{m-1} \}$ is obtained by

10 $$\begin{aligned} n = 1 + \sum _{i=1}^{m-1} 2^{m-(i+1)} [x_i > x_{i+1}] . \end{aligned}$$

In the case of $m = 4$ for example, this encoding stores the pattern $[+,+,+]$ as "1" and the pattern $[-,-,-]$ as "8" maintaining lexicographical order for the patterns in between. Analogous to the straight-forward algorithm for encoding ordinal patterns we have the same approach for difference patterns.

**Algorithm 1:**
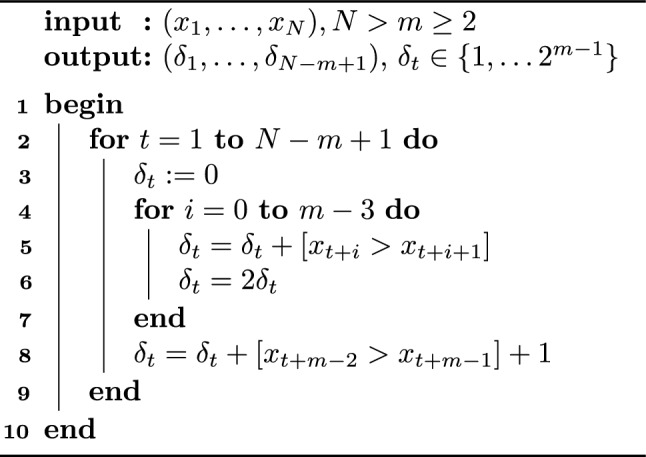
Plain Algorithm for Difference Patterns

The computational effort for one tuple is ${\mathcal {O}}(m)$, which gives for the whole time series ${\mathcal {O}}(N m)$. The detailed calculation of the computational complexity can be found in supplemental section S1. Here again, a recursive approach leads to a decrease in computational complexity. To determine the pattern of a tuple given the pattern representation of the precedent tuple, only one comparison is needed. This allows for a computational complexity that does not increase with increasing *m*, but only depends on the length of the given time series, which results in a computation time of ${\mathcal {O}}(N)$.

**Algorithm 2:**
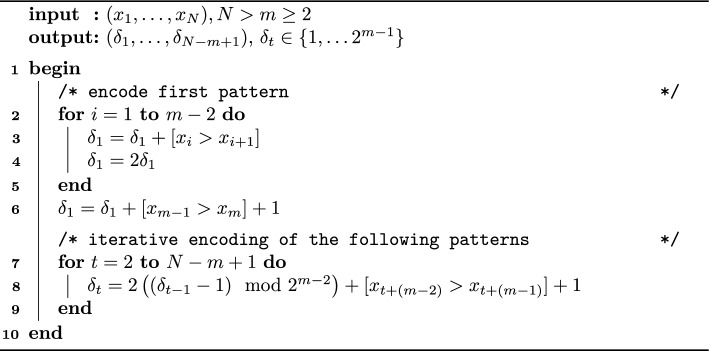
Iterative Algorithm for Difference Patterns

### Runtime analysis

First, artificial signals are used to analyze the runtime, i.e., white noise. The performances of the different encoding algorithms that were introduced in section 2.3 were evaluated by measuring the runtime of their MATLAB implementations on simulated uniform white noise where each ordinal pattern is equally likely. Using the MATLAB function rand, a vector with length $14.4 \cdot 10^6$ of uniformly distributed random numbers between 0 and 1 was generated, and the mean of 20 runs was considered. The MATLAB default *Mersenne Twister* with seeds from 1 to 20 was used for the random number generator.

Second, runtime measurements were taken for calculating EoD and PeEn on real EEG data. For this purpose, the EEG recordings of the CAP Sleep Database [19] were used, which are described in section 2.1. The EEG recordings were cut to match with the labeling of the sleep phases provided in the database. The entropic parameters were computed on windows of 6000 data points which is equivalent to 30 s.

The actual runtime performance of an algorithm depends, in general, on the platform and programming language. All measurements were taken on a laptop with 16GB RAM, an Intel Core i7-1360P processor, with operating system Windows 11 Home in MATLAB version R2023a. It should be noted that the considered algorithms were only tested for MATLAB implementations. The results could be different for other platforms, for example, pre-compiled languages like C. Therefore an extension of the investigation would be useful.

Berger et al. already mentioned in 2019 the programming technique *vectorization* which is used for the implementation of the *Plain Algorithm* (**Alg.** S1) for encoding ordinal patterns in MATLAB [16]. As a result, the implementation of this algorithm outperforms the ones of the other two algorithms, which were designed to avoid unnecessary comparisons in terms of runtime for all tested orders $m=2,\ldots ,9$.

A characteristic of programming in MATLAB is that there are commands that can process all elements of an array of data without having to construct a loop over the entries of the array. By constructing appropriate arrays and performing the comparisons, multiplications and additions on these arrays, the loops in the straightforward algorithm can be avoided in the implementation.

In the case of difference patterns, another potential way to optimize the runtime is avoiding comparisons by obtaining the vector of differences between neighbours before the actual encoding, for example, with the MATLAB function diff in the case of a one-dimensional data array. When tested on simulated uniform white noise, this approach did not lead to a reduction in runtime and was not pursued further.

### Statistical analysis

An area under the receiver operating characteristic curve (AUC) of the normalized PeEn and the normalized EoD was performed to compare the performance of the parameters when distinguishing between different states of consciousness. Sleep scoring data of the CAP Sleep Database [19] and anesthesia data of a previously published study [24] were used. The AUC is equivalent to the prediction probability for dichotomous data [34]. In the setting of anesthesia level classification, it has regularly been used to evaluate the performance of entropic measures and commercial, processed EEG indices. [7] The AUC value distinguishes two classes and ranges from 0 to 1, with 0 meaning only wrong and 1 only correct predictions. No separability of any kind is given by 0.5. We used the Statistics and Machine Learning toolbox for MATLAB (The Mathworks, Natick, MA, USA) for our analysis and calculated 95% confidence intervals for the AUC value using the cross-validation method with 10 folds. Therefore, if the value 0.5 is not in the 95% confidence interval, the result is considered statistically significant on a level $p < 0.05$, as this AUC value would mean the parameter has no effect on the classification [35].

The parameter-free machine learning algorithm linear discriminant analysis (LDA) was selected for the classification. It is a supervised classifier based on multivariate normal distribution [36] and showed good discrimination (high AUC) between the two classes for sleep and anesthesia scoring, respectively. We used the MATLAB function fitcdiscr for the training. We also tested another classification algorithm [36] with logistic regression, but since it resulted in the same AUC values and confidence intervals we focused on LDA.

## Results

In this section, we present the results for the runtimes of the encoding and the computation of the entropy-based parameters, as well as the performance for distinguishing vigilance states in sleep and in anesthesia.

### Runtime durations for encoding patterns

**Fig. 3:**
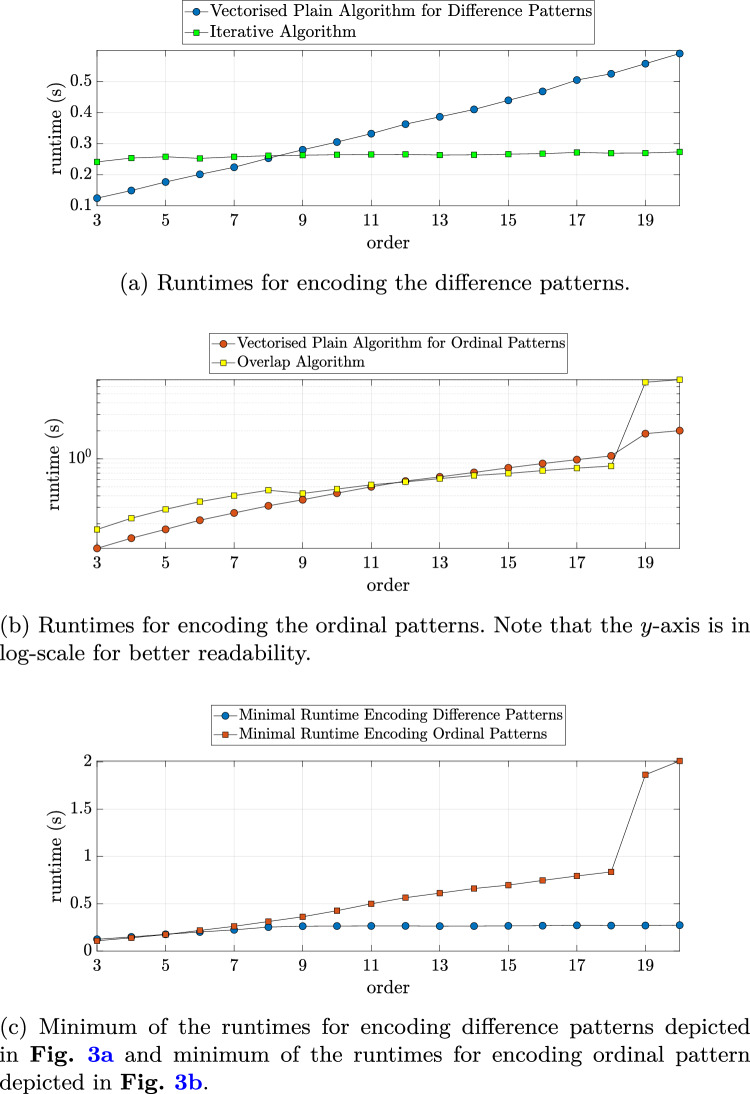
Runtimes for encoding the difference or ordinal patterns of a white noise signal of length $14.4 \times 10^6$. The figure shows the mean of 20 runs. Ties were not masked, i.e. a tie ${x}_i = {x}_j$ with $i<j$ was treated as if ${x}_i < {x}_j$

The analysis of only encoding white noise in the different patterns was done first. Fig. 3a shows that the runtime of the vectorized implementation of the *Plain Algorithm for Difference Patterns* (Alg. 1), which is increasing with embedding dimension *m* in contrast to the *Iterative Algorithm* (Alg. 2), which remained stable. The increase in duration from order $m = 3$ to order $m = 20$ was $\sim$5.5-fold for the vectorized difference pattern encoding. However, up to order 7, vectorization achieves faster runtimes in MATLAB for the plain algorithm. For the encoding of ordinal patterns the runtime increase with embedding dimension was severe for high orders. A possible explanation for the jumps visible in Fig. 3b in runtime for higher orders $m \ge 19$ is the changed numeric type as uint64 is used instead of double. This is done because of memory alignment such that an encoding of ordinal patterns is also possible for orders $m=19$ and $m=20$. For higher orders it is not possible anymore [16]. Fig. 3c shows that when choosing the optimal algorithm for each order, the encoding times for lower orders were similar for the two pattern types. For higher orders difference patterns were encoded considerably faster. At order $m = 18$, before the leap in runtime for ordinal patterns, the increase was $\sim$2.3-fold with respect to order $m = 3$ for the difference and $\sim$ 9.2-fold for the ordinal patterns.

### Runtimes of calculating entropic parameters on EEG data

**Fig. 4:**
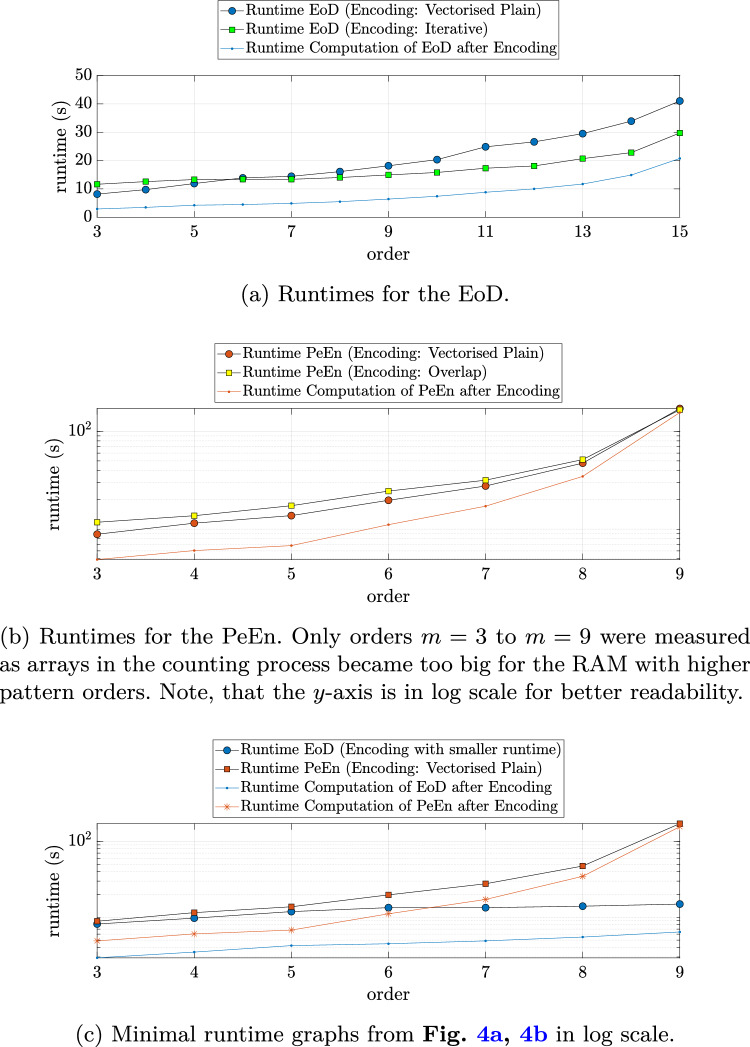
Sum of the runtimes for pattern encoding, counting the number of occurring patterns, and calculating the entropy values of 30 s windows over all 105 considered EEG recordings from the CAP Sleep Database. As before ties in the data were treated as ascending data points. The points connected by the blue and red lines indicate the total time *not* spent for encoding

For each of the EEG recordings in the CAP Sleep Database, the runtime that was needed to encode patterns, count the number of occurring patterns, and calculate the respective entropy value on 30 s windows was measured. In Fig. 4a, b, c the sum of these runtimes over all 105 considered EEG recordings is shown for the two entropic parameters and different pattern encoding options. The colored line is the time *not* spent encoding the respective patterns.

Analyzing the runtimes for EoD in Fig. 4a, one can see that for the *Iterative Algorithm*, the growth in runtime with increasing order *m* is driven by the time needed for counting patterns and computing the entropy value. This behavior is expected as the process’s runtime increases with the number of possible patterns $2^{m-1}$ while the encoding time remains constant. Compared to the measurements on the random signal in the section before, the iterative algorithm for pattern encoding outperforms the plain algorithm even sooner, namely starting from order $m=7$.

For PeEn, measurements were made up to order $m=9$, because arrays of order 10 were too large for the RAM with more than 27GB. This and the steep increase in runtime in the value counting and value computation part that is seen in Fig. 4b reflect the fact that the number of possible ordinal patterns increases much faster than the number of difference patterns, which was described in section 2.3.

Comparing the runtimes of PeEn and EoD calculation up to order $m=9$, the latter is faster than the former for all embedding dimensions, as one can see in Fig. 4c. In particular, the incline of the runtime for the pattern counting and value calculation process is much slower for EoD. The increase in these times was $\sim$2.2-fold for the EoD and $\sim$ 31.9-fold for the PeEn, as the blue and red lines show respectively.

### Sleep stages

**Fig. 5:**
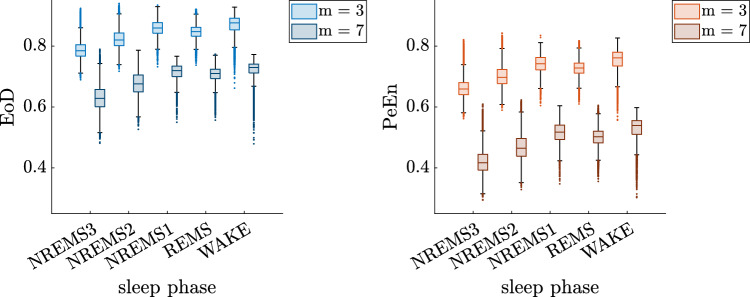
EoD and PeEn values on 30 s windows of single channel EEG for orders $m=3$ and $m=7$ respectively for all patients. The value ranges are similar for EoD and PeEn when comparing sleep phases, for higher orders the gap between the two entropic parameters gets bigger

**Fig. 6:**
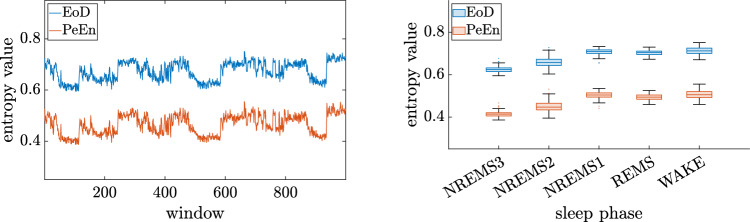
EoD and PeEn values of order $m=7$ for the patient labeled "n2" (control group, no pathology). The correlation between EoD and PeEn values of this patient is 99.53%. A similar high correlation was found across patients across orders with a light decrease for higher orders

The boxplots in Fig. 5 show the separation of the sleep phases for PeEn and EoD for different *m* for all patients. In Fig. 6 PeEn and EoD values of order $m=7$ for a single patient from the control group can be seen, a similarly high correlation is observed across all patients.

In general, EoD was considerably higher than PeEn and the level of PeEn decreased more with higher orders than for EoD, which is shown in Fig. 5. This finding was independent of any underlying disease as presented in supplemental Fig. S1. For both entropic parameters the relations of the value ranges for different phases are similar, the values for the stages WAKE and NREMS1 are on a comparable level, the other stages show a decrease from REMS to NREMS2 to NREMS3. The analysis of the 30 s EEG segments of the 105 patients at different sleep stages revealed a very high discriminative power when differentiating between wake and NREMS3 with an LDA classification performance of $\text {AUC}\ge 0.94$ for PeEn and EoD for almost all orders with, see Table 1. When differentiating between two arbitrary stages similar results were obtained regarding the comparison of PeEn and EoD, however the AUC value for both of them is lower. The detailed results are listed in supplemental section S3.2. Note that the number of windows slightly decreases with higher orders as windows containing ties were discarded and longer tuples are more likely to contain ties. Hence, the number of windows for the wake state is between 19005 and 19024 and for the NREMS3 state between 25176 and 25178. The detailed numbers of windows for each state and order are listed in supplemental Table S1. The choice of the order *m* did not considerably influence the AUC values, although higher orders showed a slight decrease in the AUC values. Randomly discarding NREMS3 windows from the dataset to balance the number of NREMS3 and wake windows led to similarly good results.

**Table 1:** AUC values with 95% confidence intervals (CoI) for an LDA classifier with 10-fold cross validation for the sleep stages Wake versus NREMS3 and orders 3 to 7. For each order the higher AUC value is highlighted with bold font

Wake vs. NREMS3		AUC (CoI)	
		PeEn	EoD
ord	3	**0.948 (0.946 0.949)**	0.948 (0.945 0.950)
	4	0.946 (0.945 0.948)	**0.947 (0.944 0.949)**
	5	**0.945 (0.943 0.947)**	0.943 (0.941 0.946)
	6	**0.944 (0.942 0.946)**	0.939 (0.937 0.942)
	7	**0.944 (0.942 0.947)**	0.936 (0.933 0.939)

### Anesthesia levels

For the anesthesia analysis, we included 14 wake EEGs, 15 during burst suppression phases, 15 from the state inter1, and 16 files from inter2 of about 120 s each and calculated PeEn and EoD over 10 s segments. As for the sleep data, windows containing ties between any data points in it were excluded from the data set. For all orders 3 to 7 there are 154 windows for the wake state. For the inter2 state there are 175 windows for order 3 and 174 for orders 4 to 7. The detailed numbers of windows for each state and order are listed in supplemental Table S2.

An analysis of an LDA classifier of wake and inter2 showed AUC values between 0.83 and 0.87 using PeEn as a predictor. Across all orders predicting with EoD led to slightly higher AUC values. In contrast to the sleep data, the AUC of PeEn decreased with higher *m* whereas for the EoD this behavior was not as distinct. Table 2 shows the detailed AUCs. When differentiating between two arbitrary stages similar results are obtained regarding the comparison of PeEn and EoD, for wake versus inter1 the values are higher, for inter1 versus inter2 they are lower. For both cases we see as well that especially the EoD has higher AUC values for higher orders. Similar results are obtained for the discrimination of each level of anesthesia for the burst suppression phase, where again the EoD exceeds the PeEn with higher AUC values for higher orders. The detailed results are listed in supplemental section S3.3.

**Table 2:** AUC values with 95% confidence intervals (CoI) for an LDA classifier with 10-fold cross validation for the stages Wake versus Inter2 and orders 3 to 7. For each order the higher AUC value is highlighted with bold font

Wake vs. Inter2		AUC (CoI)	
		PeEn	EoD
ord	3	0.861 (0.814 0.907)	**0.861 (0.819 0.903)**
	4	0.869 (0.822 0.916)	**0.875 (0.836 0.914)**
	5	0.868 (0.829 0.906)	**0.881 (0.853 0.909)**
	6	0.858 (0.809 0.907)	**0.880 (0.835 0.924)**
	7	0.839 (0.797 0.881)	**0.876 (0.847 0.904)**

**Fig. 7:**
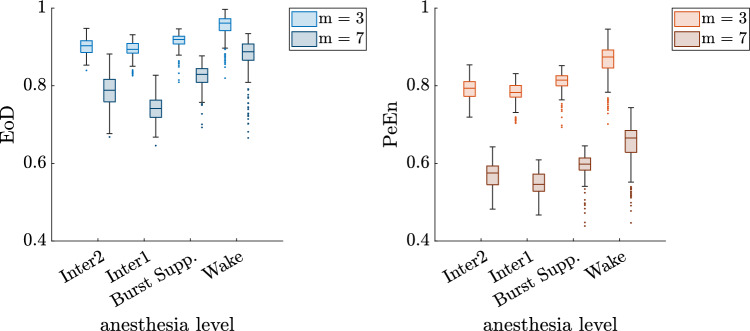
EoD and PeEn values on 10 s windows of single channel EEG for orders $m=3$ and $m=7$ respectively for all EEG recordings. The value ranges are similar for EoD and PeEn when comparing anesthesia levels, for higher orders the gap between the two entropic parameters gets bigger. Burst Supp. indicates the burst suppression level

Furthermore, the correlation of PeEn and EoD in each recording was measured. With higher orders the correlation between PeEn and EoD was considerably lower. The median of correlation valued measured was over 0.99 for order $m = 3$ but only around 0.85 for order $m = 7$. This was observed for all phases, however it was conspicuous that particularly in the wake phase considerably low correlation values were observed. The divergence of the two entropic parameters is visible in **Fig.** 7, which shows the boxplots for the entropic measures for the three different anesthesia levels and orders $m =3$ and $m=7$. The level of PeEn decreases much more with higher *m* compared to EoD. Furthermore EoD seems to better separate wake from inter1 and inter2 for higher orders. A possible explanation is that the share of ordinal patterns that actually appeared in one window in all possible ordinal patterns was much smaller with increasing order due to the shortness of the segments which led to a decrease in PeEn. In fact, starting from $m = 7$ the number of possible ordinal patterns exceeds with 5040 the used window length, which is less than a fifth of the possible patterns. The different behavior of the entropic measures can be attributed to the differing number of possible patterns.

## Discussion

With our investigation, we could show that the performance of PeEn and EoD in assessing sleep and anesthesia levels are quite similar, and the AUC values for the classification are almost the same for low orders. For higher orders the EoD can separate the levels of anesthesia better than the PeEn, as the AUC values increased. We did not observe this increase in AUC in the wake vs. NREM3 comparison, but there, the AUC values were higher from the beginning. By using the sleep and the anesthesia data, we could show that both PeEn and EoD perform well in separating different vigilance states and that there was no decrease in performance when using the computational, less demanding EoD. The analysis of the sleep stages showed an equally good performance for PeEn and EoD as for the anesthesia results. The high AUC for discriminating between NREMS3 and wake shows that the entropy measures are qualified parameters for classification. These results also justify the use of these entropic parameters in the anesthesia context as the sleep stage NREMS3 and the anesthesia level inter2 from our study show similar EEG patterns [5].

Reducing the raw EEG time series from order patterns (PeEn) to difference patterns (EoD) does not influence the discriminatory performance. While PeEn has been extensively used for analyzing brain electrical activity, especially the EEG, this has yet to be the case for EoD. A PubMed search of *"permutation entropy" AND EEG* returned 187 hits, and the search *"entropy of difference" AND EEG* returned 0 hits on August 16th 2024.

### A further reduction in information content does not affect performance

PeEn has been used in sleep and anesthesia research. Several papers highlight the possible usefulness of PeEn for sleep scoring [37–39]. In the anesthesia context, PeEn has been described to be among the best approaches to discriminate between consciousness and unconsciousness when evaluating single-channel EEG [8, 10, 12, 28]. An extension to the assessment of more than one channel by using the symbolic transfer entropy did not improve performance when compared to PeEn [40]. So, only a few pieces of information seem necessary to assess wake or sleep or conscious and unconscious levels. With EoD performing similarly to PeEn, this becomes even more obvious. The discussion about how much information entropic measures in terms of information processing really contain has been questioned in the past, especially for PeEn [14] where the authors described the relationship to the number of peaks of the raw EEG. This publication shows that PeEn calculated with $m=3$ and $\tau =1$ can be interchangeably used with "*the power spectral centroid of the signal’s first derivative and Kedem’s higher order crossings*" [14]. Kedem’s algorithm of higher order crossings [41] was described in 1986 and is based on assessing, e.g., the number of peaks. This already shows that even little information may suffice to evaluate vigilance states or anesthetic levels. PeEn seems to function for separating conscious and unconscious states in patients as shown by several groups [8, 10, 12, 40] and in this article. EoD showed a similar performance but is computationally less demanding because of a lower number of possible patterns and a simpler way of encoding, as the sign vector generation for EoD is straightforward. As shown, the computation duration depends on encoding the "difference patterns". However, the process is more straightforward than encoding ordinal patterns for PeEn, for which each new data point’s amplitude at a window shift of one point has to be put in relation to the previous $m-1$ points. There are different analytical approaches to creating these patterns [16], but the simplicity of getting the "difference" patterns seems to put EoD at an advantage. When using EoD, the need for a temporal component, i.e., the time point of a certain amplitude value being essential to define the order, becomes obsolete as the reduction to "$+$" or "−" does not require this information. With our analyses, we could show that a further reduction of the recorded EEG from ordinal patterns to merely "$+$" and "−", i.e., a binary vector, does not reduce performance in assessing vigilance states.

In a more scientific context, the reduction in analyzed information content highlights that assessing vigilance states with the EEG does not seem to require a focus on complex features but can be done on simple binary vectors. Other binary methods like the zero-crossing rate [42, 43] or the Lempel-Ziv complexity [44, 45] perform as well for sleep and anesthesia. Because the published studies on PeEn and anesthesia use rather low embedding dimensions [8, 10], one could argue that our investigation of the run times for high embedding dimensions is unimportant. But there may be something to it.

High embedding dimensions have the advantage of better separation of consciousness and unconsciousness in the anesthesia setting for $m = 7$ up until $m=9$ [8]. Baseline situations with eyes open and eyes closed scenarios are also separated very well by the PeEn using these orders [46, 47]. It is suggested that even higher orders *m* should be used when tied values are involved in the signal if the computational costs and memory requirements suffice [31]. The EoD can help here, as it has been shown that higher orders are possible with the same signal length, and the computational complexity is lower. New methods, such as the analysis of non-occurring patterns, are possible for higher embedding dimensions. They can help separate the consciousness states, e.g., in anesthesia, as the non-occurring patterns seem an even better indicator for the different levels [15]. Our analyses are based on EEG signals filtered to rather low frequencies below 30 Hz to reduce the impact of muscle activity in the form of EMG contamination, [21]. Still, we know that EMG covers the entire EEG range, and even resting state EMG can influence the EEG [48]. The PeEn appears to be most sensitive to the higher frequencies in a filtered signal when using low orders *m*. At higher orders, the cutoff frequency for the lowpass filter can be set higher as the classification improves [8]. Commercial EEG-based monitoring systems also seem to focus on the faster frequencies when calculating the index. A recent paper elegantly describes the focus of the bispectral index (BIS) on low gamma-band activity [49].

At higher embedding dimensions, the PeEn decreases in a seemingly paradoxical fashion. This behavior can be attributed to the number of non-occurring ordinal patterns. For PeEn the number of possible patterns is *m*!. Because typically the EEG episodes used for anesthesia monitoring are around 10 s and the sample rate probably not higher than 1 kHz, *m*! quickly exceeds the number of data points in an episode. For EoD the number of possible patterns grows more slowly. So for higher *m* we do not see the paradoxical behavior for EoD that strongly.

### Clinical implications

The so-called depth of anesthesia monitoring, as it has been applied over the last decades, focuses on the hypnotic component of anesthesia. This means that the monitoring is based on an index that is matched onto a one dimensional scale. But as anesthesia navigation may not be one dimensional, i.e. not behave like a submarine [50], the monitoring approaches need to be extended to at least a two-dimensional plane that also considers the analgesic component of anesthesia [51]. The design of one parameter that can cover both entities may be tricky and maybe composite indices would provide an easier solution. PeEn and EoD may be suitable components for the hypnotic component. In contrast to spectral approaches, both can directly be applied to the raw signal without the need to transform it to the frequency-domain. PeEn’s strength to separate consciousness from unconsciousness has been reported [8, 10, 12] and mentioned earlier. More recent research also suggests that PeEn, in contrast to spectral measures, does not follow the paradoxical excitation, which is an EEG activation pattern, predominately in the beta-band of the EEG [5] that causes typical, spectral indices to indicate an "increased level of wakefulness". PeEn in contrast monotonously decreased during this "excitation phase" [52]. This indicates that the onset of the strong beta oscillatory activity is an initial phase of the transition into anesthetic-induced unconsciousness which is reflected by lower PeEn caused by synchronization processes which may indicate less information processing.

Reducing the computational time of an algorithm can be relevant in the clinical setting because a patient monitor during general anesthesia must process multiple physiological parameters and tasks simultaneously, resulting in longer computational times for each. Therefore, faster algorithms with comparable performance may be a suitable option for integration.

### Limitations

Of course, this investigation has several limitations. All analyses were retrospective and the entire topic was of rather technical nature. So far no entropic, time-domain measure is used for patient monitoring. But these entropic approaches can help to better understand the EEG dynamics and have proven useful as research tool. We also only focused on the hypnotic component as mentioned above. Future EEG-patient monitoring approaches should follow a more holistic approach that includes other information like, e.g., nociception. In these approaches the entropic measures may be useful for assessment of hypnosis. Especially at higher *m* and for the awake state, PeEn and EoD seem to diverge and show low correlation coefficients, as depicted in supplemental Fig. S2. The reason, therefore, most probably lies in the algorithm. First, the higher the *m*, the more rank order patterns can be coded into the same sign pattern. Second, with higher *m*, the percentage of non-occurring patterns increases, especially for PeEn. This leads to a divergence between PeEn and EoD. In our analyses, we focused on EEG recorded during steady states to show the comparable performance of EoD when compared to PeEn. The next step of the performance assessment needs to be the evaluation of EoD and PeEn behavior during transitions between the vigilance states or between the anesthesia levels.

## Conclusion

A further reduction of analyzed EEG information, i.e. from amplitude order patterns to amplitude sign patterns does not compromise the performance of entropic parameters to reliably distinguish between wakefulness and anesthetic-induced unconsciousness. On the contrary, for higher orders the EoD can do so better than the PeEn, especially when short window lengths are considered.

## Supplementary Information

Below is the link to the electronic supplementary material.

**Supplementary information:** The MATLAB code used for encoding of the difference patterns is available online at \url{https://gitlab.tuwien.ac.at/alexander.edthofer/entropycalculation}. Additional information is also given a supplementary PDF-file. (442 KB)
