# Supplementary material for: Entropy of difference works similarly to permutation entropy for the assessment of anesthesia and sleep EEG despite the lower computational effort
Source: J Clin Monit Comput. 2024 Dec 26;39(4):655–68. doi: 10.1007/s10877-024-01258-8 (PMC12304019; doi:10.1007/s10877-024-01258-8)
Supplement: Supplementary file 1 — Supplementary information: The MATLAB code used for encoding of the difference patterns is available online at \url{https://gitlab.tuwien.ac.at/alexander.edthofer/entropycalculation}. Additional information is also given a supplementary PDF-file. (442 KB) [file 10877_2024_1258_MOESM1_ESM.pdf]

Supplementary information

Entropy of Difference works Similarly to Permutation Entropy for the Assessment of Anesthesia and Sleep EEG despite the Lower Computational Effort

S1 Complexity of the algorithms

S1.1 Encode ordinal patterns with plain algorithm

Algorithm S1: Plain Algorithm for Ordinal Patterns[16]

input : $(x_1, \ldots, x_N), N > m \geq 2$
output: $(\pi_1, \ldots, \pi_{N-m+1}), \pi_t \in \{0, \ldots, m! - 1\}$

1 **begin**
2   **for** $t = 1$ **to** $N - m + 1$ **do**
3     $\pi_t := 0$
4   **for** $i = 0$ **to** $m - 2$ **do**
5   **for** $j = i + 1$ **to** $m - 1$ **do**
6         $\pi_t = \pi_t + [x_{t+i} > x_{t+j}]$
7   **end**
8       $\pi_t = (m - i)\pi_t$
9   **end**
10   **end**
11 **end**

For $i = 2, \ldots, 10$ the amount of time that is needed to execute line i is denoted by c_i. It is assumed that the `end` commands do not require any effort, i.e. $c_7 = c_9 = c_{10} = 0$. The run-time amounts to

$$T(N, m) = (N - m + 2)\, c_2 + (N - m + 1)\, T_{\text{inner}}(m),$$

where

$$\begin{aligned} T_{\text{inner}}(m) &= c_3 + m c_4 \\ &\quad + \sum_{i=0}^{m-2} \left(c_8 + (m - i)\, c_5 + (m - (i + 1))\, c_6\right) \\ &= \left(\frac{c_5}{2} + \frac{c_6}{2}\right) m^2 + \left(c_4 + \frac{c_5}{2} - \frac{c_6}{2} + c_8\right) m \\ &\quad + c_3 - c_5 - c_8. \end{aligned}$$

The time needed to compute one pattern is $T_{\text{inner}}(m)$ and is in $\mathcal{O}(m^2)$.

$$T(N,m) = c_2(N-m) + 2c_2 + (N-m)(am^2 + bm + c) \\ + am^2 + bm + c$$

Therefore, $T(N,m)$ is in $\mathcal{O}((N-m)m^2)$. As $N \gg m$, it follows that $T(N,m) = \mathcal{O}(Nm^2)$.

S1.2 Encode ordinal patterns with overlap algorithm

Algorithm S2: Overlap Algorithm for Ordinal Patterns[16]

```
   input  : (x_1, ..., x_N), N > m ≥ 2
   output: (π_1, ..., π_{N−m+1}), π_t ∈ {0, ... m! − 1}
1  begin
2      for j = 1 to m do
3          r_j := 0
4      end
       /* obtain initial right inversion counts                */
5      for i = 1 to m − 2 do
6          for j = i + 1 to m − 1 do
7              r_{i+1} = r_{i+1} + [x_i > x_j]
8          end
9      end
       /* encode the following patterns recursively            */
10     for t = 1 to N − m + 1 do
11         π_t := 0
12         for j = 1 to m − 1 do
13             r_j = r_{j+1} + [x_{t+j−1} > x_{t+m−1}]
14             π_t = (m − j)(π_t + r_j)
15         end
16     end
17 end
```

The runtime for one line is again denoted by c_i for $i = 2, \ldots, 17$ with the assumption that the `end` commands do not require any time.

$$T(N,m) = (m+1)c_2 + mc_3 + T_{\text{first}}(m) + T_{\text{following}}(N,m),$$

The summands T_{first} and $T_{\text{following}}$ consist of

$$\begin{aligned}T_{\text{first}}(m) &= (m-1)c_5 + \sum_{i=1}^{m-2} (m-i)c_6 + (m-(i+1))c_7 \\ &= \left(\frac{c_6}{2} + \frac{c_7}{2}\right) m^2 + \left(c_5 - \frac{c_6}{2} + \frac{c_7}{2}\right) m \\ &\quad - c_5 - c_6 - 3c_7,\end{aligned}$$

and

$$\begin{aligned}T_{\text{following}}(N,m) = (N-m+2)\, c_{10} + (N-m+1) \\ (c_{11} + mc_{12} + (m-1)(c_{13} + c_{14}))\,.\end{aligned}$$

Accordingly, T_{first} is in $\mathcal{O}(m^2)$ and $T_{\text{following}}$ in $\mathcal{O}((N-m)m)$, which results for $T(N,m)$ in $\mathcal{O}(\max(m^2,(N-m)m))$. As $N \gg m$, it follows that $T(N,m) = \mathcal{O}(Nm)$.

S1.3 Encode difference patterns with plain algorithm

The run-time with the assumption from above is

$$T(N,m) = \ (N-m+2)c_2 + (N-m+1)T_{\text{inner}}(m),$$

where

$$T_{\text{inner}}(m) = (c_3 + c_8) + (m-1)c_4 + (m-2)(c_5 + c_6)$$

is the time needed to encode one pattern. T_{inner} is in $\mathcal{O}(m)$, therefore the run-time T is in $\mathcal{O}((N-m)m)$, which simplifies to $\mathcal{O}(Nm)$.

S1.4 Encode difference patterns with iterative algorithm

$$T(N,m) = T_{\text{first}}(m) + T_{\text{following}}(N,m)$$

with

$$T_{\text{first}}(m) = (m-1)c_2 + (m-2)(c_3 + c_4) + c_6 \in \mathcal{O}(m)$$

and

$$\begin{aligned}T_{\text{following}}(N,m) &= (N-m+1)c_7 + (N-m)c_8 \\ &\in \mathcal{O}(N-m).\end{aligned}$$

The run-time $T(N,m)$ is in $\mathcal{O}(\max(m,(N-m)))$, which is in $\mathcal{O}(N)$ because $N \gg m$.

S2 Supplemental figures

S2.1 Sleep stages

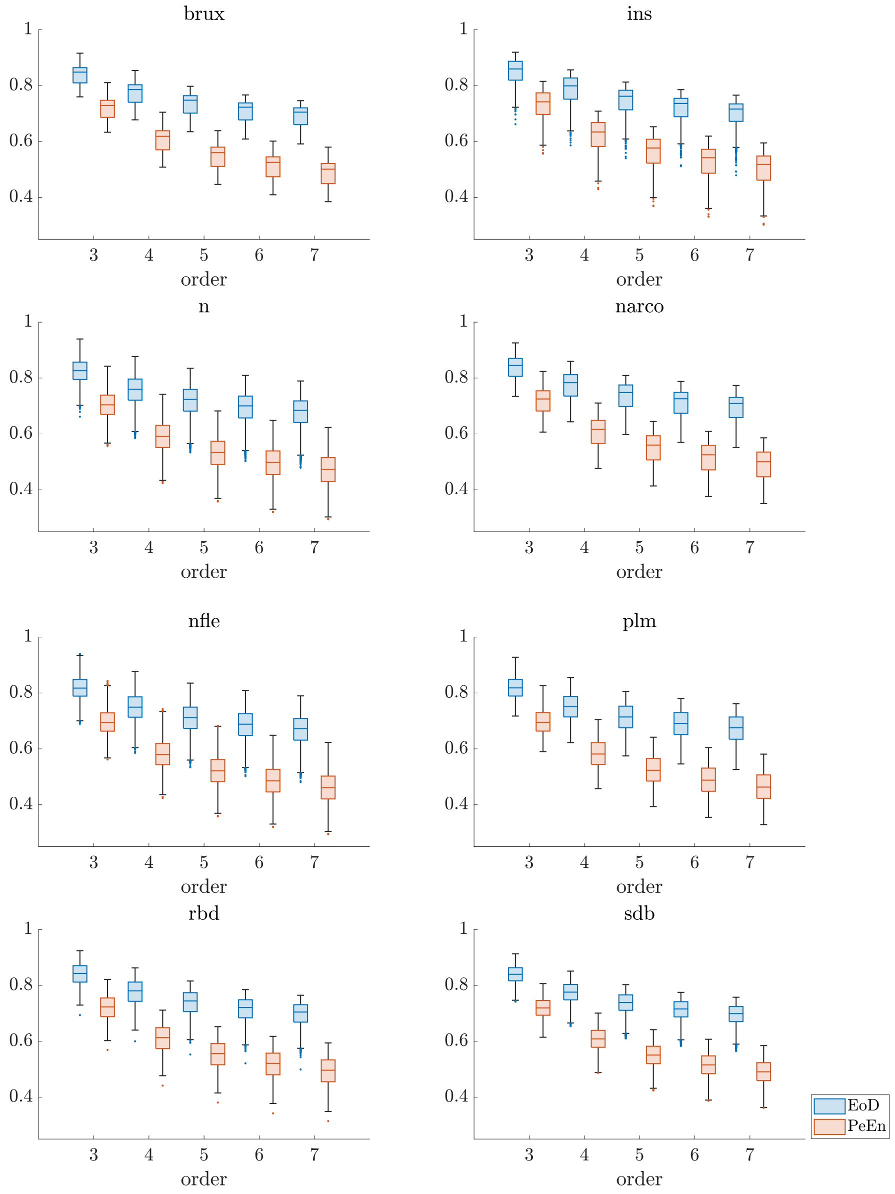

Fig. S1: Range of PE and EoD broken down between all the diseases. The number of patients for each disorder is listed in the original publication.[19]

S2.2 Anesthesia levels

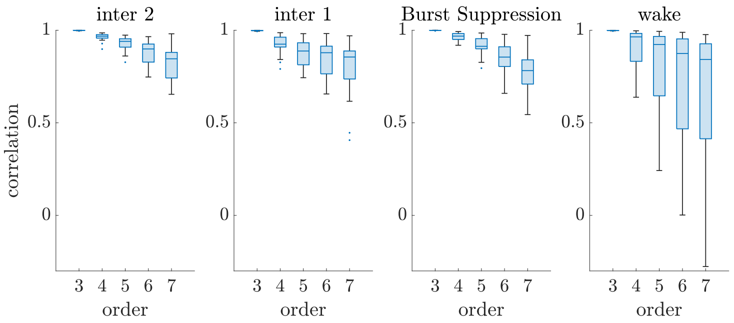

Fig. S2: For each of the available recordings for each anesthesia level the correlation between PeEn and EoD values was evaluated on 10 s windows. These numbers, that were obtained for orders $m = 3$ to $m = 7$, are summarized with boxplots.

S3 Supplemental tables

S3.1 Number of windows for each dataset

ord	number of windows				
	wake	REMS	NREMS1	NREMS2	NREMS3
3	19024	18520	4578	38266	25178
4	19021	18520	4578	38265	25178
5	19018	18520	4578	38262	25176
6	19015	18520	4578	38262	25176
7	19005	18519	4577	38254	25176

Table S1: Number of windows of each state for each order in the CAP sleep database.

ord	number of windows			
	wake	inter1	inter2	Burst Supp.
3	154	165	175	165
4	154	165	174	165
5	154	165	174	165
6	154	165	174	165
7	154	165	174	165

Table S2: Number of windows of each state for each order in the anesthesia dataset. Burst Supp. indicates the level burst suppression.

S3.2 AUC tables for each pair of stages of consciousness for the CAP sleep database

Wake vs. NREMS2		AUC (CoI) PeEn	EoD
ord	3	**0.867 (0.865 0.869)**	0.867 (0.863 0.870)
	4	**0.866 (0.863 0.869)**	0.866 (0.863 0.869)
	5	**0.864 (0.861 0.868)**	0.861 (0.857 0.865)
	6	**0.863 (0.860 0.866)**	0.852 (0.848 0.855)
	7	**0.863 (0.859 0.867)**	0.841 (0.838 0.844)

Table S3: AUC values with 95% confidence intervals (CoI) for an LDA classifier with 10-fold cross validation for the sleep stages Wake versus NREMS2 and orders 3 to 7. For each order the higher AUC value is highlighted with bold font.

Wake vs. NREMS1		AUC (CoI) PeEn	EoD
ord	3	**0.645 (0.637 0.653)**	0.644 (0.635 0.654)
	4	**0.646 (0.638 0.654)**	0.645 (0.639 0.652)
	5	**0.644 (0.634 0.658)**	0.638 (0.631 0.646)
	6	**0.642 (0.632 0.652)**	0.625 (0.616 0.634)
	7	**0.642 (0.638 0.646)**	0.609 (0.599 0.618)

Table S4: AUC values with 95% confidence intervals (CoI) for an LDA classifier with 10-fold cross validation for the sleep stages Wake versus NREMS1 and orders 3 to 7. For each order the higher AUC value is highlighted with bold font.

Wake vs. REMS		AUC (CoI) PeEn	EoD
ord	3	**0.756 (0.749 0.762)**	0.756 (0.750 0.761)
	4	0.754 (0.749 0.759)	**0.754 (0.750 0.759)**
	5	**0.750 (0.746 0.755)**	0.744 (0.737 0.751)
	6	**0.748 (0.745 0.752)**	0.723 (0.717 0.728)
	7	**0.750 (0.746 0.754)**	0.700 (0.695 0.705)

Table S5: AUC values with 95% confidence intervals (CoI) for an LDA classifier with 10-fold cross validation for the sleep stages Wake versus REMS and orders 3 to 7. For each order the higher AUC value is highlighted with bold font.

REMS vs. NREMS3		AUC (CoI) PeEn	EoD
	3	**0.914 (0.911 0.916)**	0.914 (0.911 0.917)
	4	0.911 (0.908 0.915)	**0.912 (0.909 0.915)**
**ord**	5	**0.910 (0.907 0.913)**	0.910 (0.907 0.913)
	6	**0.910 (0.907 0.913)**	0.908 (0.906 0.910)
	7	**0.911 (0.909 0.912)**	0.907 (0.904 0.909)

Table S6: AUC values with 95% confidence intervals (CoI) for an LDA classifier with 10-fold cross validation for the sleep stages REMS versus NREMS3 and orders 3 to 7. For each order the higher AUC value is highlighted with bold font.

REMS vs. NREMS2		AUC (CoI) PeEn	EoD
	3	0.738 (0.734 0.742)	**0.738 (0.735 0.741)**
	4	**0.739 (0.736 0.742)**	0.739 (0.734 0.744)
**ord**	5	**0.740 (0.737 0.744)**	0.740 (0.736 0.744)
	6	0.740 (0.736 0.744)	**0.741 (0.737 0.745)**
	7	0.739 (0.733 0.744)	**0.741 (0.736 0.745)**

Table S7: AUC values with 95% confidence intervals (CoI) for an LDA classifier with 10-fold cross validation for the sleep stages REMS versus NREMS2 and orders 3 to 7. For each order the higher AUC value is highlighted with bold font.

REMS vs. NREMS1		AUC (CoI) PeEn	EoD
	3	0.635 (0.624 0.646)	**0.635 (0.630 0.640)**
	4	0.629 (0.624 0.635)	**0.630 (0.616 0.643)**
**ord**	5	**0.624 (0.616 0.631)**	0.620 (0.615 0.625)
	6	**0.622 (0.615 0.629)**	0.605 (0.596 0.614)
	7	**0.624 (0.614 0.635)**	0.595 (0.585 0.605)

Table S8: AUC values with 95% confidence intervals (CoI) for an LDA classifier with 10-fold cross validation for the sleep stages REMS versus NREMS1 and orders 3 to 7. For each order the higher AUC value is highlighted with bold font.

NREMS1 vs. NREMS3		AUC (CoI)	
		PeEn	EoD
	3	**0.929 (0.926 0.932)**	0.929 (0.925 0.933)
	4	0.927 (0.923 0.930)	**0.927 (0.925 0.930)**
**ord**	5	**0.925 (0.922 0.928)**	0.924 (0.921 0.927)
	6	**0.924 (0.922 0.927)**	0.921 (0.918 0.924)
	7	**0.925 (0.922 0.929)**	0.919 (0.914 0.923)

Table S9: AUC values with 95% confidence intervals (CoI) for an LDA classifier with 10-fold cross validation for the sleep stages NREMS1 versus NREMS3 and orders 3 to 7. For each order the higher AUC value is highlighted with bold font.

NREMS1 vs. NREMS2		AUC (CoI)	
		PeEn	EoD
	3	0.803 (0.797 0.809)	**0.803 (0.799 0.808)**
	4	0.801 (0.796 0.806)	**0.801 (0.795 0.808)**
**ord**	5	**0.799 (0.794 0.804)**	0.797 (0.789 0.805)
	6	**0.798 (0.791 0.804)**	0.791 (0.785 0.796)
	7	**0.798 (0.793 0.803)**	0.786 (0.781 0.791)

Table S10: AUC values with 95% confidence intervals (CoI) for an LDA classifier with 10-fold cross validation for the sleep stages NREMS1 versus NREMS2 and orders 3 to 7. For each order the higher AUC value is highlighted with bold font.

NREMS2 vs. NREMS3		AUC (CoI)	
		PeEn	EoD
	3	0.799 (0.796 0.802)	**0.799 (0.796 0.802)**
	4	0.793 (0.788 0.797)	**0.795 (0.792 0.798)**
**ord**	5	0.789 (0.786 0.791)	**0.790 (0.786 0.794)**
	6	**0.788 (0.785 0.791)**	0.786 (0.782 0.790)
	7	**0.790 (0.787 0.793)**	0.783 (0.779 0.786)

Table S11: AUC values with 95% confidence intervals (CoI) for an LDA classifier with 10-fold cross validation for the sleep stages NREMS2 versus NREMS3 and orders 3 to 7. For each order the higher AUC value is highlighted with bold font.

S3.3 AUC tables for each pair of stages of consciousness for the anesthesia dataset

Wake vs. Inter1		AUC (CoI) PeEn	EoD
	3	**0.880 (0.841 0.920)**	0.880 (0.838 0.921)
	4	0.893 (0.860 0.925)	**0.899 (0.871 0.927)**
**ord**	5	0.897 (0.853 0.941)	**0.920 (0.898 0.941)**
	6	0.895 (0.863 0.928)	**0.928 (0.899 0.957)**
	7	0.876 (0.838 0.915)	**0.925 (0.887 0.963)**

Table S12: AUC values with 95% confidence intervals (CoI) for an LDA classifier with 10-fold cross validation for the stages Wake versus Inter1 and orders 3 to 7. For each order the higher AUC value is highlighted with bold font.

Inter1 vs. Inter2		AUC (CoI) PeEn	EoD
	3	0.611 (0.538 0.683)	**0.611 (0.556 0.666)**
	4	0.662 (0.601 0.723)	**0.663 (0.619 0.708)**
**ord**	5	0.689 (0.614 0.765)	**0.731 (0.694 0.768)**
	6	0.693 (0.630 0.755)	**0.770 (0.733 0.807)**
	7	0.685 (0.642 0.728)	**0.789 (0.760 0.818)**

Table S13: AUC values with 95% confidence intervals (CoI) for an LDA classifier with 10-fold cross validation for the stages Inter1 versus Inter2 and orders 3 to 7. For each order the higher AUC value is highlighted with bold font.

Wake vs. Burst Suppression		AUC (CoI) PeEn	EoD
	3	0.833 (0.787 0.879)	**0.833 (0.789 0.878)**
	4	0.841 (0.800 0.882)	**0.843 (0.805 0.882)**
**ord**	5	0.834 (0.770 0.897)	**0.841 (0.812 0.870)**
	6	0.821 (0.773 0.869)	**0.838 (0.786 0.892)**
	7	0.802 (0.760 0.844)	**0.834 (0.777 0.890)**

Table S14: AUC values with 95% confidence intervals (CoI) for an LDA classifier with 10-fold cross validation for the stages Wake versus Burst Suppression and orders 3 to 7. For each order the higher AUC value is highlighted with bold font.

**Inter1 vs. Burst Suppression**		**AUC (CoI)** PeEn	EoD
	3	**0.823 (0.772 0.873)**	0.822 (0.775 0.869)
	4	0.873 (0.838 0.907)	**0.882 (0.859 0.905)**
**ord**	5	0.889 (0.860 0.919)	**0.923 (0.897 0.950)**
	6	0.887 (0.840 0.934)	**0.939 (0.900 0.978)**
	7	0.870 (0.814 0.927)	**0.950 (0.927 0.973)**

Table S15: AUC values with 95% confidence intervals (CoI) for an LDA classifier with 10-fold cross validation for the stages Inter1 versus Burst Suppression and orders 3 to 7. For each order the higher AUC value is highlighted with bold font.

**Inter2 vs. Burst Suppression**		**AUC (CoI)** PeEn	EoD
	3	**0.711 (0.667 0.755)**	0.707 (0.629 0.786)
	4	0.729 (0.690 0.768)	**0.737 (0.684 0.790)**
**ord**	5	0.727 (0.647 0.807)	**0.753 (0.712 0.793)**
	6	0.725 (0.656 0.795)	**0.765 (0.706 0.824)**
	7	0.712 (0.673 0.751)	**0.775 (0.749 0.802)**

Table S16: AUC values with 95% confidence intervals (CoI) for an LDA classifier with 10-fold cross validation for the stages Inter2 versus Burst Suppression and orders 3 to 7. For each order the higher AUC value is highlighted with bold font.
